# Chemistry and pharmacology of anticancer drugs

**DOI:** 10.1038/sj.bjc.6604075

**Published:** 2007-12-11

**Authors:** R A Sikes

**Affiliations:** 1 The University of Delaware, DE, USA

*Chemistry and Pharmacology of Anticancer Drugs* by David E Thurston has tackled a mountain of information that exists regarding the current state of cancer chemotherapy. The sheer volume of information published on both cancer and chemotherapeutics is frequently discouraging to novice cancer researchers and often turns them away from investigations on the action or activity of new drugs alone or in the combination. This book is written in a style that is easily understood and fairly comprehensive without delving into elaborate dosing tables and various regimens for delivery. As written, the book does an excellent job for its intended target audience of advanced undergraduates and health-care professionals in training or those with only peripheral exposure to cancer therapy.

The book begins with a very brief introduction to cancer that includes: basic terminology of cancer and metastasis, staging and grading, treatment options, evaluation of chemotherapeutics, limiting toxicity and mechanisms of chemotherapy that are sufficient but will not impress the professional cancer researcher or clinician. However, the information is presented in a manner that will appeal to professionals or students who desire a rapid overview in this area. After this introduction, the book discusses chemotherapeutic compounds by class, in a manner reminiscent of more clinically rich clinical oncology texts: anti-metabolites, DNA interactive, anti-tubulin, molecular pathway targets and hormonal pathway targets. The author discusses each compound of every class; the class of compound is followed by detailed descriptions of the structure and mechanism of each drug in its class. Toxicities, side effects and current proscribed uses are included, but only as used in the United Kingdom. For those readers who are interested in a source that compiles chemotherapeutics by class and mechanism in a single table, keep waiting. This is one big deficiency of the text. Typically, these tables were provided with administration routes and scheduling information that made them too cumbersome for all but the clinical user. The author has missed a major opportunity to simplify a summary presentation.

After this, the book takes a turn towards an examination of more novel and experimental therapies, something usually not found in books that are targeted at the advanced undergraduate to early graduate student level. Essentially, the book handles topics on tumour targeting strategies using antibodies and antivascular approaches as well as bioreductive agents. This trend is continued in the chapter on biological agents, which includes information on biological response modifiers and modulation of the immune system including vaccines. The book then delves into treatments on the horizon that are based upon fairly recent basic and translational research. The author provides a very nice overview of the number of strategies and the specificity for targeting cancer resistance, growth factor signalling pathways and the integration of genomics and proteomic responses for tailoring or determining if treatments are successful. This is followed-up in a later chapter on personalised medicine, which also includes a section on predicting adverse effects to chemotherapy. This section concludes with a brief overview of chemoprevention strategies and the most favoured chemoprevention agents that are used currently; the section also includes a discussion, or at least a speculation, of their mechanism of action. The book concludes with a section on adjunct therapies to ease major side effects and improve patient tolerance of therapy.

It is a minor source of irritation that once drugs have been discussed at length they are no longer page referenced in the text and are referred to only by the trade name and not by the compound name, which is how they are listed most commonly in the index. This has the reader referring to the table of contents and the individual drug sections to try to refresh their memory of drugs as they come back into discussion in the text through a different context. Better cross-listing of compounds and a more complete index is needed but is not a major distraction.

In short, the back jacket description is quite accurate and the book does not disappoint given the target audience. Finding drugs through the table of contents or through the index is sufficient. Overall, the book is easy to read and manages to not be redundant. This is all quite an accomplishment considering the topic.

